# Supplementary material for: Anti-seizure medication tapering correlates with daytime delta band power reduction in the cortex
Source: Brain Commun. 2025 Feb 25;7(1):fcaf020. doi: 10.1093/braincomms/fcaf020 (PMC11851006; doi:10.1093/braincomms/fcaf020)
Supplement: fcaf020_Supplementary_Data [file fcaf020_supplementary_data.pdf]

Anti-seizure medication tapering correlates with daytime delta band power reduction in the cortex

Supplematary Materials

Guillermo M. Besné[1], Nathan Evans[1], Mariella Panagiotopoulou[1], Billy Smith[1], Fahmida A Chowdhury[3], Beate Diehl[3], John S Duncan[3], Andrew W McEvoy[3], Anna Miserocchi[3], Jane de Tisi[3], Matthew Walker[3], Peter N. Taylor[1,2,3], Chris Thornton[1], Yujiang Wang[1,2,3*]

January 13, 2025

1. CNNP Lab (www.cnnp-lab.com), Interdisciplinary Computing and Complex BioSystems Group, School of Computing, Newcastle University, Newcastle upon Tyne, United Kingdom

2. Faculty of Medical Sciences, Newcastle University, Newcastle upon Tyne, United Kingdom

3. UCL Queen Square Institute of Neurology, Queen Square, London, United Kingdom

* Yujiang.Wang@newcastle.ac.uk

Supplementary Material Section 1 Subject and ASM details

N	22
Age (mean±SD)	31.1±8.6
Sex (M:F)	8:14
Epilepsy (Temporal:Frontal)	16:6
Side (Left:Right)	10:12
Num icEEG contacts (mean±SD)	71.1±27.0
Num ROIs (mean±SD)	16.3±5.6
Days of recording (mean±SD)	8.1±5.0
Num of regular ASM (mean±SD)	2.5±0.9
Num of tapered ASM (mean±SD)	2.3±0.9

Supplementary Table 1.1: Summary of subject data used in the analysis.

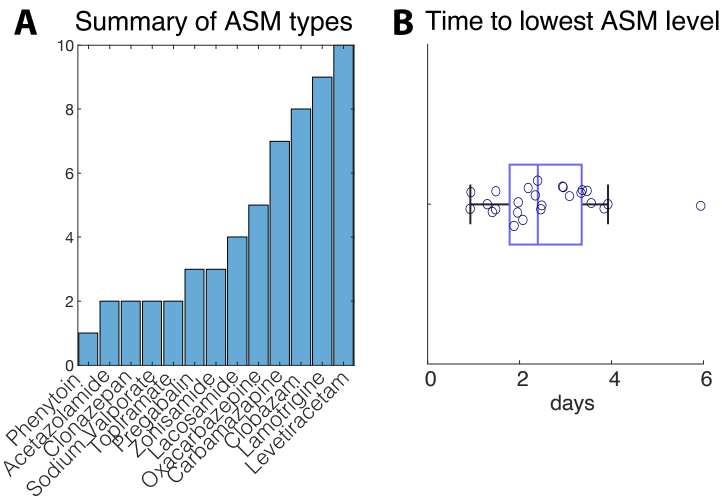

Supplementary Figure 1.1: **A** Histogram of number of subjects (N=22) receiving each Anti-Seizure Medication (ASM, N=13) type. **B** Distribution of the time between the start of medication tapering and reaching the minimum ASM plasma levels ($avr = 2.45days$, N=22).

Supplementary Material Section 2 Plasma concentration modelling with first-order pharmaco-kinetics

As reported in the *Methods*, we used pharmaco-kinetic modelling to better represent the effects of ASMs on the electrophysiology. Here, Supplementary Equation 1 is used to model the effect on plasma concentration of a single ASM intake with a first-order absorption and elimination.

$$C(t) = \frac{F * D * Ka}{Vd * (Ka - Ke)} * (e^{\text{-Ke*t}} - e^{\text{-Ka*t}}) \quad (1)$$

Equation 1: Pharmaco-kinetic equation for plasma concentration of a single dose oral intake with first-order absorption and elimination. *C(t)*, plasma concentration. *F*, bio-availability. *D*, dose at intake. *Ka*, absorption constant. *Ke*, elimination constant. *t*, time since intake. *Vd*, volume distribution.

In this equation several metabolising and plasma availability parameters are considered, exclusive for each ASM type. Considering all subjects included in our dataset, there are 13 different ASMs, listed in Supplementary Figure 1.1A and Supplementary Table 2.1. Bio-availability (F) represent the proportion of ASM that reaches circulation, and volume distribution (Vd) assist on the conversion from dose to concentration. This two parameters represent the availability of the digested medication, while the dynamics of ASM metabolising are represented by elimination (Ke) and absorption (Ka) constants as part of a exponential. Most parameters have been obtained from DB; Iapadre et al. (2018); Patsalos (2022) considering the parameters of each medication from a single site. However, Ke and Ka had to be estimated from 2 other parameters: time to maximum concentration (T_{max}) and elimination half-life ($t_{1/2}$, time required to eliminate half the concentration).

ASM	T_{max}	$t_{1/2}$	Ka	Ke	F	Vd	T_{max}(Solver)
Levetiracetam	1.30	7.0	2.618	0.099	1.00	0.60	1.30
Lamotrigine	3.10	55.5	1.572	0.012	0.98	1.10	3.10
Clobazam	1.25	32.0	4.244	0.022	1.00	1.43	1.25
Carbamazapine	6.00	14.5	0.403	0.048	0.80	1.00	6.00
Oxacarbazepine	4.50	9.0	0.487	0.077	1.00	0.70	4.50
Zonisamide	4.00	63.0	1.180	0.011	0.95	1.45	4.00
Sodium Valproate	4.00	11.0	0.644	0.063	0.90	0.16	4.00
Pregabalin	1.50	6.3	2.065	0.110	0.90	0.50	1.50
Lacosamide	1.00	13.0	4.486	0.053	1.00	0.60	1.00
Topiramate	3.05	21.0	1.215	0.033	0.80	0.70	3.05
Clonazepan	2.50	60.0	2.091	0.012	0.90	3.00	2.50
Acetazolamide	3.00	12.5	1.029	0.055	0.90	0.30	3.00
Phenytoin	8.00	22.0	0.322	0.032	1.00	0.75	8.00

Supplementary Table 2.1: Pharmaco-kinetic parameters for ASMs included in this study. *F*, bio-availability. *Ka*, absorption constant. *Ke*, elimination constant. $t_{1/2}$, elimination half-life. *Vd*, volume distribution. T_{max}, time to maximum plasma concentration. T_{max}*(Solver)*, time to maximum plasma concentration estimated by Microsoft Excel *Solver* and Supplementary Equation 2

.

Ke estimation was calculated as: $ln(2)/t_{1/2}$. The estimation of Ka was obtained by resolving

Supplementary Equation 2. Given its complexity, an optimisation method was applied: Microsoft Excel *Solver*. *Solver* adjusts one or several cells to match a formula cell to a specific target. In our case, we have our formula of Supplementary Equation 2 as well as the values of Ke and T_{max} obtained from the literature. For a specific ASM, we defined a new cell, T_{max}(Solver), as the result of implementing Supplementary Equation 2 using cells for Ke and Ka. Then, *Solver* tool is configured to modify the value of Ka and match the value of T_{max}(Solver) with T_{max}. Once the process is finished, the new values of Ka and T_{max}(Solver) are automatically stored. This process is repeated individually for each ASMs.

$$T_{\max} = \frac{ln(\frac{Ka}{Ke})}{Ka - Ke} \tag{2}$$

Equation 2: Pharmaco-kinetics equation to estimate time to maximum plasma concentration of a single dose oral intake with first order absorption and elimination. T_{max}, time to maximum plasma concentration. Ka, absorption constant. Ke, elimination constant.

The result from Supplementary Equation 1 is the influence of a single ASM intake on plasma concentration. To model the continuous intakes and tapering for a specific subject, we combined several modelled influences, modifying the dose and the time of intake to match clinical reports. To complete the plasma concentration modelling, the steady-state concentration has to be modelled as well. The steady-state is reached after a prolonged and continuous ASM intake, and the required time changes between drugs. We approached this task by extending the intake schedule for a long period - 2 months before icEEG recordings started - following the regular treatment of each individual. Once modelled, the extra period is removed.

Supplementary Material Section 3 ASM tapering effect on canonical frequency bands

In addition to the δ frequency band, we also explored the effect of ASM tapering on the other canonical frequency bands: Theta (θ: 4-8 Hz), Alpha(α: 8-13 Hz), Beta (β: 13-30 Hz) and Gamma (γ: 30-47.5 Hz, 52.5-57.5 Hz, 62.5-77.5 Hz). Following the same analysis presented in the main text, we measured the change in $\log_{10}$ power of each frequency band between the pre-tapered

period and the tapered period. To better understand these changes, we also obtained the change in relative band power, as the contribution of each frequency band to the sum of the $\log_{10}$ band power of each canonical frequency bands.

From the data presented in Supplementary Figure 3.1 and Supplementary Table 3.1, we can determine that the ASM tapering is generating an overall band power reduction with a spectral shift. Band power change is strongest for δ and gradually weakens as the frequency increases, showing no change for γ. Wilcoxon signed rank test confirms this behaviour - δ to β negative size effects are observed with a strong significance. In contrast, changes in γ show small size effects with no significance. In terms of relative band power, from δ to α a reduction is observed (only significant on δ), while β and γ show a significant increase in relative band power. These two findings combined reveal that there is an overall reduction of band power on top of the spectral shift, where high frequency bands are less affected due to tapering than low frequency bands.

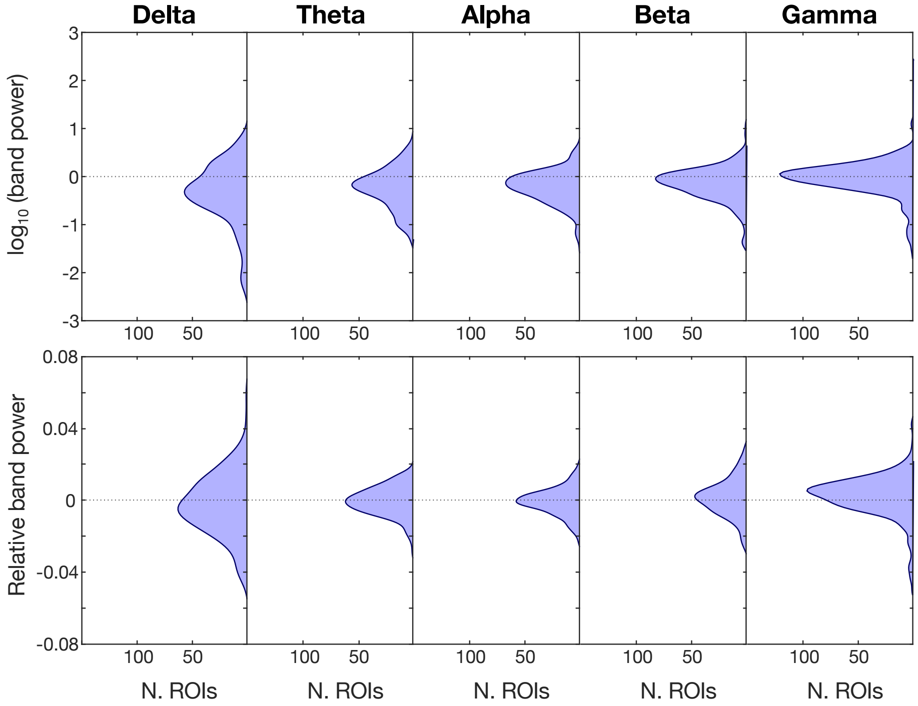

Supplementary Figure 3.1: Distribution of delta (1-4Hz), theta (4-8Hz)), alpha (8-13Hz)), beta (13-30Hz) and gamma (30-47.5Hz, 52.5-57.5Hz, 62.5-77.5Hz) band power (Top Row) and relative band power (Bottom Row) changes due to tapering for all Regions Of Interest (ROIs, N=372 for all frequency bands) and subjects (N=22 for all frequency bands). Further statistical analyses are presented below in Supplementary Table 3.1.

		δ	θ	α	β	γ
$\log_{10}$ Band Power	Eff. Size	-0.489	-0.505	-0.558	-0.418	-0.037
	p-Value	<0.001	<0.001	<0.001	<0.001	0.484
Relative Band Power.	Eff. Size	-0.148	-0.029	-0.084	0.123	0.311
	p-Value	0.0047	0.582	0.120	0.019	<0.001

Supplementary Table 3.1: Effect size and p-value of Wilcoxon signed rank test for band power (Top Row) and relative band power (Bottom Row) changes due to tapering on δ (delta, 1-4Hz), θ (theta, 4-8Hz)), α (alpha, 8-13Hz)), β(beta, 13-30Hz) and γ (gamma, 30-47.5Hz, 52.5-57.5Hz, 62.5-77.5Hz) frequency bands

Supplementary Material Section 4 Hierarchical model predicting band power changes

We used hierarchical modelling throughout our study to assist us in confirming some of our findings while considering the influence of confounding factors. We built several hierarchical models as mixed-effect linear models with a common framework: δ $\log_{10}$ band power changes in each ROI ($\triangle\delta$) are predicted, based on a random-effect for individual subjects (ID), and modifying the fixed-effects to test each case.

4.1 Confirming effects of tapering strength and its regional differences

Adjusting for subject-effect using the mixed-effects model confirmed the effects of tapering strength and its regional differences. We fitted two mixed-effect linear models with fixed effects for: A) the relative percentage of the lowest ASM level reached during tapering (minASM): $\triangle\delta \sim 1 +$ minASM + (1|ID), Supplementary Table 4.1 1st panel; B) the interaction between minASM and the classification of each ROI as cortical or sub-cortical (CORT): $\triangle\delta \sim 1 +$minASM*CORT + (1|ID), Supplementary Table 4.1 2nd panel.

The fitted models confirm most of our findings with the Wilcoxon signed rank test and Spearman's rank correlation, but refutes the dose-dependant effect observed on sub-cortical regions. As observed in Supplementary Table 4.1 2nd panel, only the cortical region shows an overall decrease of δ band power and a dose-dependent effect on it.

We also explored further parcellation of cortical regions dividing it into lobes (Lobe): Frontal,

Cingulates, Parietal, Temporal and Occipital: $\triangle\delta \sim 1$ + minASM*Lobe + (1|ID), Supplementary Table 4.1 3rd panel.The resulting model reveals that all cortical regions have similar decrease of δ band power and a dose-dependent behaviour.

Model	Fixed-Effect	Estimate	p-Value	CI(Lower)	CI(Upper)
minASM	minASM	1.377	0.008	0.352	2.401
minASM*CORT	Inter.(Sub-cortical)	-0.499	0.194	-1.254	0.256
	minASM	0.709	0.219	-0.424	1.843
	Cortical	-0.835	<0.001	-1.243	-0.428
	minASM:Cortical	0.755	0.012	0.161	1.35
minASM*Lobe	Inter.(Occipital)	-0.980	0.026	-1.845	-0.116
	Frontal	-0.688	0.039	-1.341	-0.036
	Cingulate	-0.165	0.692	-0.982	0.652
	Temporal	-0.147	0.632	-0.749	0.455
	Parietal	-0.729	0.015	-1.316	-0.142
	minASM	0.953	0.016	-0.362	2.269
	minASM:Frontal	1.050	0.042	0.036	2.065
	minASM:Cingulate	0.372	0.558	-0.876	1.62
	minASM:Temporal	0.201	0.668	-0.72	1.121
	minASM:Parietal	0.984	0.034	0.072	1.896

Supplementary Table 4.1: Table showing the coefficient estimates and p-values of the fixed-effect predictors for the following mixed-effect linear models from top to bottom: **1)** $\triangle\delta \sim 1$ + minASM + (1|ID); **2)** $\triangle\delta \sim 1$ +minASM*CORT + (1|ID); **3)** $\triangle\delta \sim 1$ + minASM*Lobe + (1|ID). "$\triangle\delta$": change in delta frequency band power, "CORT": label indicating Cortical or Sub-cortial region, "Lobe": label indication if the region corresponds to Occipital, Frontal, Cingulate, Temporal or Parietal lobes. Predictors "CORT" and "Lobe" are categorical values, therefore intercept (Inter) represent the category on brackets.

4.2 Seizure onset, frequently-spiking or later surgically resected regions do not have characteristic behaviour

In addition to regional differences the effects of ROIs being within the seizure onset zone (SOZ), later surgically resected areas (RSC), or frequently-spiking (SPK), have also been studied. We labelled each ROI as SOZ, RSC or SPK based on the number of channels within these categories applying thresholds of 1 channel for SOZ and 25% for RSC and SPK. We fit the independent mixed-effect models with a fixed effect for the interaction between each label and cortical parcellation ($\triangle\delta \sim 1$ + SOZ*CORT + (1|ID)), to determine if there are behavioural changes on SOZ, RSC or SPK areas. Supplementary Table 4.2 shows that seizure onset zones, surgically resected areas and

spiking regions show no distinctive behaviour compared to the rest of the tissue.

Model	Fixed-Effect	Estimate	p-Value	CI(Lower)	CI(Upper)
SOZ*CORT	Inter.(Sub-cortical & nSOZ)	-0.091	0.513	-0.366	0.183
	SOZ	0.035	0.765	-0.193	0.262
	Cortical	-0.330	<0.001	-0.509	-0.152
	SOZ:Cortical	0.046	0.721	-0.208	0.301
RSC*CORT	Inter.(Sub-cortical & nRSC)	-0.022	0.878	-0.303	0.259
	RSC	-0.073	0.524	-0.301	0.153
	Cortical	-0.398	<0.001	-0.589	-0.208
	RSC:Cortical	0.107	0.397	-0.141	0.355
SPK*CORT	Inter.(Sub-cortical & nSPK)	-0.082	0.670	-0.461	0.296
	SPK	0.0141	0.934	-0.321	0.349
	Cortical	-0.336	0.036	-0.649	-0.023
	SPK:Cortical	0.014	0.936	-0.328	0.356

Supplementary Table 4.2: Table showing the coefficient estimates and p-values of the fixed-effect predictors for the following mixed-effect linear models from top to bottom: **1)** $\triangle\delta \sim 1 + \text{SOZ*CORT} + (1|\text{ID})$; **2)** $\triangle\delta \sim 1 + \text{RSC*CORT} + (1|\text{ID})$; **3)** $\triangle\delta \sim 1 + \text{SPK*CORT} + (1|\text{ID})$. "$\triangle\delta$": change in delta frequency band power, "CORT": label indicating Cortical or Sub-cortial region, "SOZ": label indicating Seizure Onset Zone regions, "SPK": label indicating regularly spiking regions, "RSC": label indicating later surgically resercted regions. Predictors "CORT", "SOZ", "SPK" and "RSC" are categorical variables, therefore intercept (Inter.) represent the combination of categories on brackets.

4.3 Similar behaviour among ASM classes

As reported in the literature, different ASMs have specific effects on each of the canonical frequency bands. Our low sample size and the heterogeneity of ASM use during tapering makes studying individual medication effects unreliable. Therefore, we classified ASMs based on their primary physiological target. As an example, Clobazam and Clonazepam are classified as "GABA-ergic" (GB) due to mostly affecting gabba receptors, while Carbazapine and Oxarbazapine as "Sodium Channels" (SC). All ASM are classified into 5 groups: GABA-ergic (GB), Sodium Channel (SC), SV2A receptor (SV), Multi-targel (ML) and Other Targets (OT, including ASM classifications with low representations). As ASMs belonging to multiple classes can be tapered at the same time, each one is assigned a binary variable and the intercept is removed as all subjects that had at least 1 ASM tapered - a simplification will be used going forward where ASM_c = SC+ML+GB+SV+OT-1. The fitted model captures the effect of each ASM class and the reduction of ASM plasma

concentration level reached with them (minASM). We only considered cortical ROIs for this analysis, as sub-cortical regions show no significant changes due to tapering. The resulting model ($\triangle\delta \sim 1 + ASM_c$*minASM + (1|ID), Supplementary Table 4.3) indicates that subjects tapered with Multi-Target ASM have stronger overall δ band power reduction but no dose-dependency.

Model	Fixed-Effect	Estimate	p-Value	CI(Lower)	CI(Upper)
minASM*ASM_c	minASM	-0.481	0.463	-1.766	0.805
	SC(t)	-0.217	0.831	-2.213	1.779
	ML(t)	-2.128	0.087	-4.566	0.31
	GB(t)	-0.324	0.727	-2.146	1.498
	SV(t)	-1.341	0.183	-3.318	0.636
	OT(t)	1.640	0.451	-2.631	5.91
	minASM:SC(t)	0.429	0.767	-2.418	3.276
	minASM:ML(t)	3.394	0.09	-0.536	7.324
	minASM:GB(t)	0.289	0.846	-2.636	3.214
	minASM:SV(t)	2.036	0.197	-1.059	5.131
	minASM:OT(t)	-2.260	0.493	-8.743	4.223

Supplementary Table 4.3: Table showing the coefficient and p-values of the fixed-effect predictors for mixed-effect linear model $\triangle\delta \sim 1 + ASM_c$*minASM + (1|ID). "$\triangle\delta$": change in delta frequency band power, "minASM": minimum modelled Anti-Seizure Medication (ASM) levels, "ASM_c": ASM class based on physiological target, GABA-ergic (GB), Sodium Channel (SC), SV2A receptor (SV), Multi-targel (ML) and Other Targets (OT, including ASM classifications with low representations)

4.4 Recovery time from surgery does not affect band power changes

All subjects included in our study underwent surgery for icEEG electrode implantation. Several factors related to the surgery and its recovery have the potential of affect the icEEG recordings: recovery from anaesthesia, inflammatory processes, stress of a prolonged hospitalisation and others. Therefore, we built a hierarchical model to determine if the duration of time between surgery to tapering has an effect on the observed band power changes. This time gap (SGRt) is defined as the time between the day of the surgery and the end of baseline-ASM period - the first instance when tapering effects are observed. The selected definition for SGRt prevents its interaction with the minimum ASM level reached during tapering (minASM) when the hierarchical model is fitted.

The applied hierarchical model captures the effects of these two parameters and regional differences between cortical and sub-cortical regions ($\triangle\delta \sim 1$ + (minASM+SRGt)*CORT + (1|ID),

Supplementary Table 4.4) and reveals that a longer time since surgery does not have a significant effect on δ band power changes.

Model	Fixed-Effect	Estimate	p-Value	CI(Lower)	CI(Upper)
(minASM+SRGt)*CORT	Inter.(Sub-cortical)	-0.476	0.250	-1.288	0.336
	minASM	0.569	0.322	-0.559	1.697
	SRGt	0.012	0.837	-0.093	0.115
	Cortical	-1.054	<0.001	-1.548	-0.56
	Cortical:minASM	0.637	0.040	0.029	1.245
	Cortical:SRGt	0.056	0.12	-0.015	0.128

Supplementary Table 4.4: Table showing the coefficient estimates and p-values of the fixed-effect predictors for mixed-effect linear $\triangle\delta \sim 1$ + (minASM+SRGt)*CORT + (1|ID) applied on tapered individuals. "$\triangle\delta$": change in delta frequency band power, "minASM": minimum modelled Anti-Seizure Medication (ASM) levels, "SRGt": time between surgery and ASM tapering beginning, "CORT": label indicating Cortical or Sub-cortial region. Predictors "CORT" is a categorical variable, therefore intercept (Inter.) represent the category in brackets.

4.5 Not ASM tapered cohort

In addition to tapered subjects, we have also investigated a sample of icEEG monitoring subjects that did not undergo ASM tapering. These subjects were experiencing seizures regularly even on full ASM dose, making tapering unnecessary. This likely reflects either a difference in disease aetiology or in other factors such as the stress of surgery that may have lowered seizure threshold. This, combined with differences in proximity to surgery, and icEEG recording duration, makes direct comparisons between tapered and not tapered subjects difficult. However, here we have attempted to make accommodations, allowing us to include these subjects in a limited analysis.

To reflect that these individuals did not go through ASM tapering and were constantly at 'full-dose', the minimum ASM plasma concentration level was set to 1. To obtain the 24 h time windows of baseline-ASM and reduced-ASM, we attempted to replicate the distribution of the time distances between surgery, start of tapering and minimum ASM levels on tapered subject. First, we obtained these time distances (surgery to start of tapering, and start of tapering to minimum ASM) for all tapered subjects, and extracted the mean, standard deviation, maximum and minimum values. These values are used to generate random time distance values that fit on the distribution defined by the mean and standard deviation, and keeping them within the

maximum and minimum values. However, because the overall length of the recordings is shorter for non-tapered subjects, the time between surgery, baseline-ASM, and reduced-ASM is shorter than for the tapered subjects.

We added 10 not tapered subjects to our previous 22 tapered subjects and reproduced the hierarchical model presented in Supplementary Material Section 4.4. The resulting model (Supplementary Table 4.5) showed no substantial change compared to the model fitted with only tapered subjects (Supplementary Table 4.4). The similarity between models consolidates our findings, showing a consistent dose-effect while including subject on 'full-dose'.

Giving the similarity between models, the complications introduced to the methodology by shorter recordings, and a likely difference in severity of the disease, we did not investigate the non-tapered subjects further.

Model	Fixed-Effect	Estimate	p-Value	CI(Lower)	CI(Upper)
(minASM+SRGt)*CORT	Inter.(Sub-cortical)	-0.082	0.863	-1.014	0.850
	minASM	-0.712	0.143	-1.665	0.242
	SRGt	0.070	0.220	-0.042	0.183
	Cortical	-1.032	<0.001	-1.609	-0.456
	Cortical:minASM	0.542	0.072	-0.050	1.135
	Cortical:SRGt	0.062	0.111	-0.014	0.139

Supplementary Table 4.5: Table showing the coefficient estimates and p-values of the fixed-effect predictors for mixed-effect linear model $\triangle\delta \sim 1 + (\text{minASM+SRGt})*\text{CORT} + (1|\text{ID})$ applied on tapered and non tapered individuals. "$\triangle\delta$": change in delta frequency band power, "minASM": minimum modelled Anti-Seizure Medication (ASM) levels, "SRGt": time between surgery and ASM tapering beginning, "CORT": label indicating Cortical or Sub-cortial region. Predictors "CORT" is a categorical variable, therefore intercept (Inter.) represent the category in brackets.

References

Drug bank online. `https://go.drugbank.com`.

Giulia Iapadre, Ganna Balagura, Luca Zagaroli, Pasquale Striano, and Alberto Verrotti. Pharmacokinetics and drug interaction of antiepileptic drugs in children and adolescents. *Pediatr Drugs*, page 429–453, 2018. doi: 10.1007/s40272-018-0302-4.

Philip N. Patsalos. *Antiseizure Medication Interactions - A Clinical Guide*. Springer, 2022.
